# Supplementary material for: Molecular basis for the bifunctional Uba4–Urm1 sulfur‐relay system in tRNA thiolation and ubiquitin‐like conjugation
Source: EMBO J. 2020 Sep 9;39(19):e105087. doi: 10.15252/embj.2020105087 (PMC7527816; doi:10.15252/embj.2020105087)
Supplement: Supplementary file 1 — Appendix [file EMBJ-39-e105087-s001.pdf]

Appendix

Molecular basis for the bifunctional Uba4-Urm1 sulfur relay system in tRNA thiolation and ubiquitin-like conjugation

Marta Pabis[1,#], Martin Termathe[2,#], Keerthiraju E. Ravichandran[1,3,#], Sandra D. Kienast[2,4], Rościsław Krutyhołowa[1,5], Mikołaj Sokołowski[1,3], Urszula Jankowska[1], Przemysław Grudnik[1], Sebastian A. Leidel[2,4,*] and Sebastian Glatt[1,*]

[1] Malopolska Centre of Biotechnology (MCB), Jagiellonian University, Krakow, Poland

[2] Max Planck Institute for Molecular Biomedicine, Von-Esmarch-Str. 54, 48149 Muenster, Germany.

[3] Postgraduate School of Molecular Medicine, Warsaw, Poland

[4] Department of Chemistry and Biochemistry, University of Bern, Freiestrasse 3, 3012 Bern, Switzerland

[5] Faculty of Biochemistry, Biophysics and Biotechnology, Jagiellonian University, Krakow, Poland.

# these authors contributed equally

* Correspondence to SAL (sebastian.leidel@dcb.unibe.ch) and SG (sebastian.glatt@uj.edu.pl)

Key words: thiolation, tRNA modification, adenylation, UBL, thioester

Running title: Structure and Function of Uba4

Table of Contents

Appendix Figures

- S1 - Multiple sequence alignment of eukaryotic and prokaryotic homologues of *Ct*Uba4.
- S2 - Analyses of *Ct*Uba4 surface properties.
- S3 - Yeast control experiments.
- S4 - SEC profiles.

Appendix Tables

- S1 - Thermostability of *Ct*Uba4 proteins.
- S2 - Activity assays of all *Ct*Uba4 mutants *in vitro*.
- S3 - Yeast strains used in this study
- S4 - Results of mass spectrometry analysis of Urm1 conjugation site.

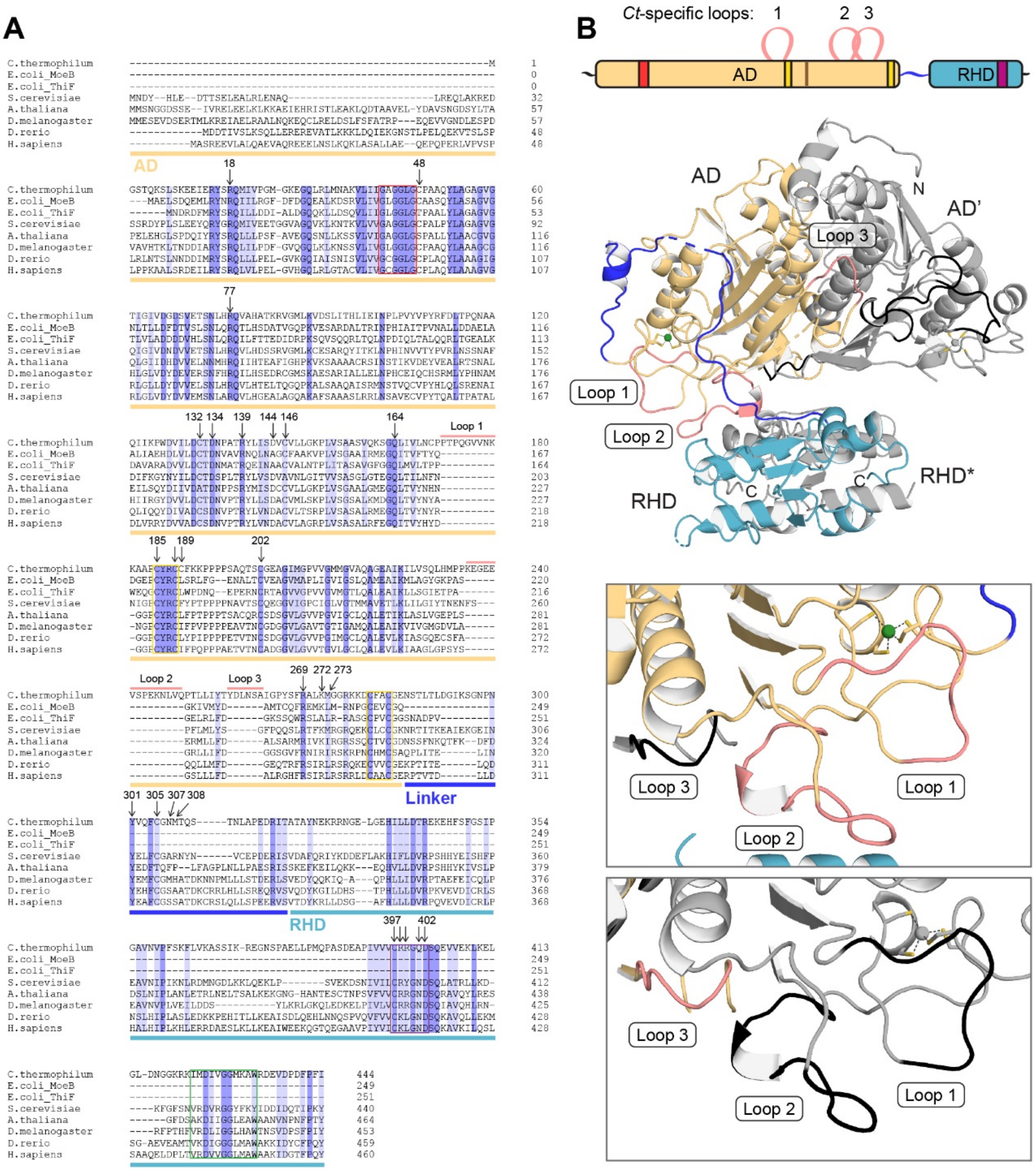

C

*Ct*Uba4	*Sc*Uba4 (Uniprot ID: P38820)	*Hs*MOCS3 (Uniprot ID: O95396)
Identity	40.6 %	37.1 %
Similarity	67.7 %	63.6 %

*Ct*Urm1	*Sc*Urm1 (Uniprot ID: P40554)	*Hs*Urm1 (Uniprot ID: Q9BTM9)
Identity	46.1 %	48.5 %
Similarity	76.5 %	77.2 %

Appendix Figure S1. Multiple sequence alignment of eukaryotic and prokaryotic homologues of *Ct*Uba4. (A) Sequence alignment of *Ct*Uba4 with selected eukaryotic and prokaryotic homologues. Completely conserved residues (dark blue) and residues that are exchanged by a similar residue (light blue) are highlighted. Boxed areas indicate the conserved active loops in the AD (red) and RHD (purple), the Cys-X-X-Cys zinc ion coordination (yellow) and the rhodanese domain signature (green) motifs. *Ct*-specific sequences are marked by pink lines. (B) Schematic representation of the *Ct*Uba4 domain organization with indication of the localization of *Ct*-specific loops. Cartoon representation of the *Ct*Uba4 structure with *Ct*-specific loops colored in pink and black. Zoomed views of loops of each *Ct*Uba4 molecule from the asymmetric unit. (C) Summary of sequence identity and similarity between *Ct* proteins and their eukaryotic homologues.

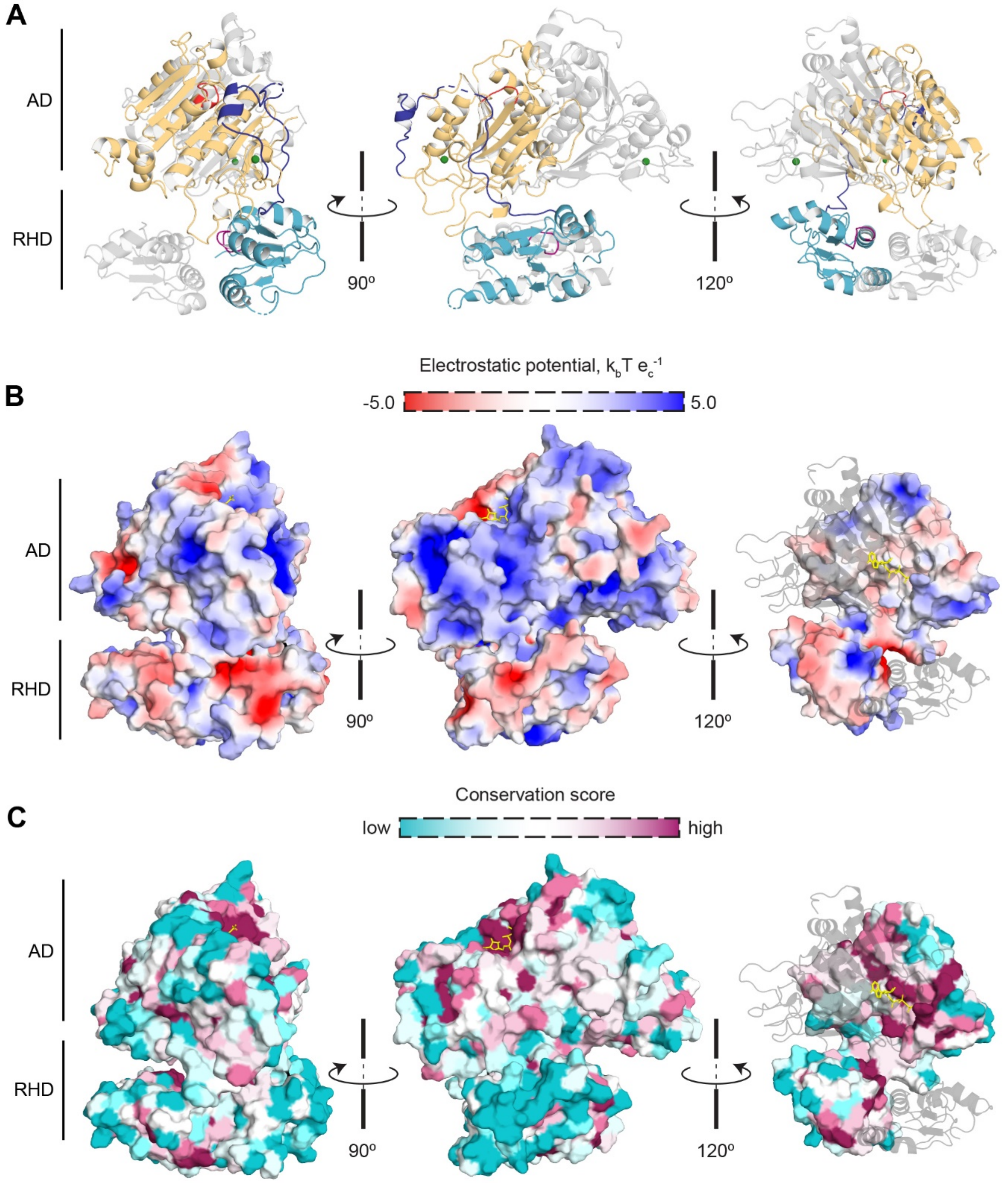

Appendix Figure S2. Analyses of *Ct*Uba4 surface properties. (A) Cartoon representation of the *Ct*Uba4 homodimer indicating the AD (yellow) and RHD (cyan). (B) Surface electrostatic potential of the *Ct*Uba4 homodimer (left and middle) and the dimerization interface (right). (C) Analysis of surface conservation of Uba4 using sequences from five fungi (*Saccharomyces cerevisiae, Candida glabrata, Schizosaccharomyces pombe, Aspergillus niger, Chaetomium thermophilum*), five plants (*Arabidopsis thaliana, Nicotiana tabacum, Physcomitrella patens subsp. Patens, Zea mays, Oryza sativa subsp. japonica*) and five animals (*Danio rerio, Gallus gallus, Bos Taurus, Mus musculus, Homo sapiens*). Surface conservation of the *Ct*Uba4 homodimer (left and middle) and the dimerization interface (right). (B-C) ATP (yellow) was positioned within the nucleotide-binding pocket according to the ATP bound *Ec*MoeB-MoaD structure (PDB ID: 1JWA).

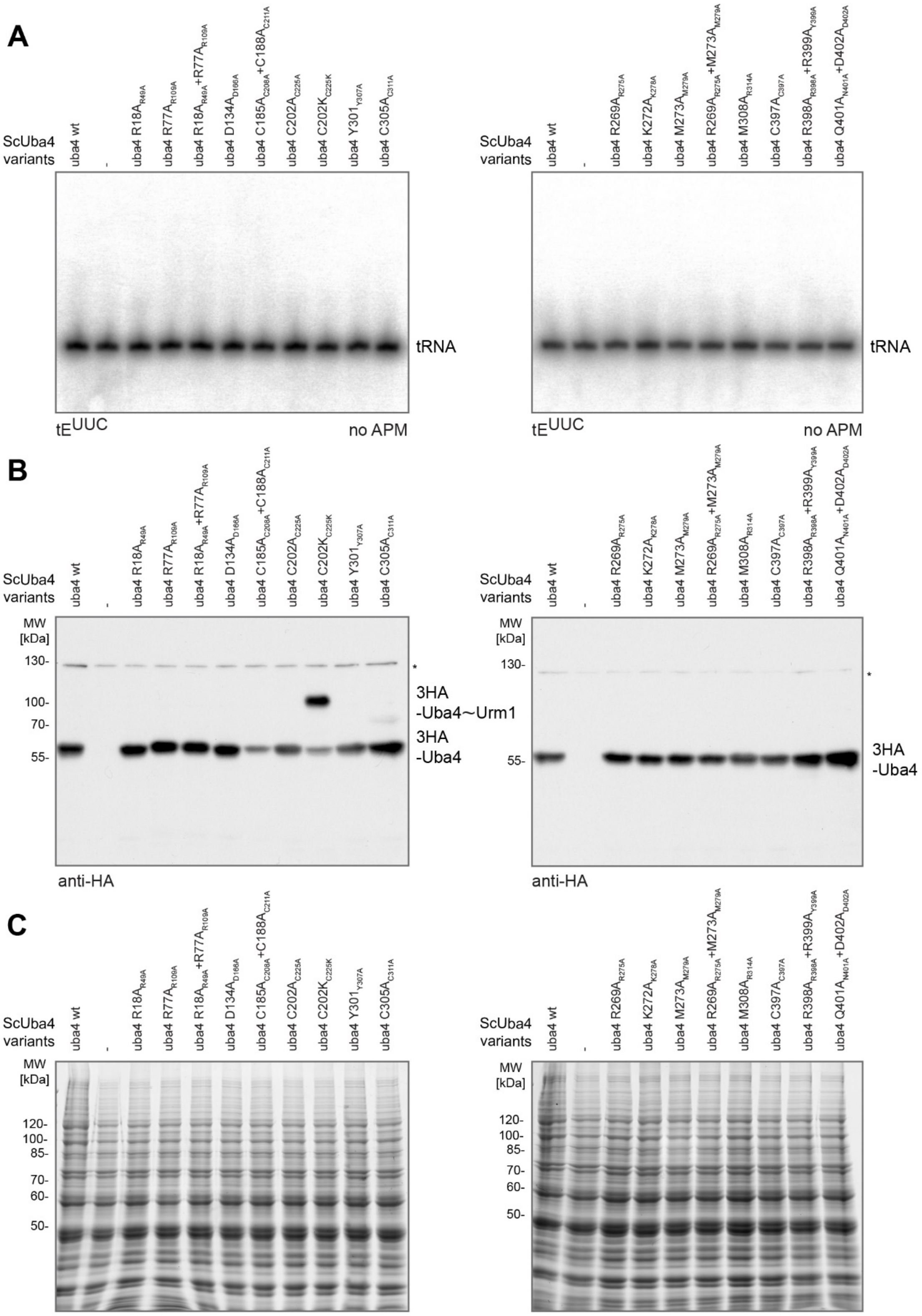

Appendix Figure S3 Yeast control experiments. (A) Northern blot analysis of tRNA on a standard PAGE. Control gel to show equal RNA extraction efficiency and loading. (B) Western blot analysis to investigate expression pattern of different 3HA-tagged *Sc*Uba4 mutants. Asterisk (*) indicates an unspecific signal. (C) Analysis of total protein extracts from different *Sc*Uba4 mutant yeast strains used in b on a separate SDS-PAGE. Control gel to demonstrate equal protein extraction efficiency and loading. All residue numbering follows *Ct*Uba4 sequence, but the respective *Sc*Uba4 numbering is added in subscript.

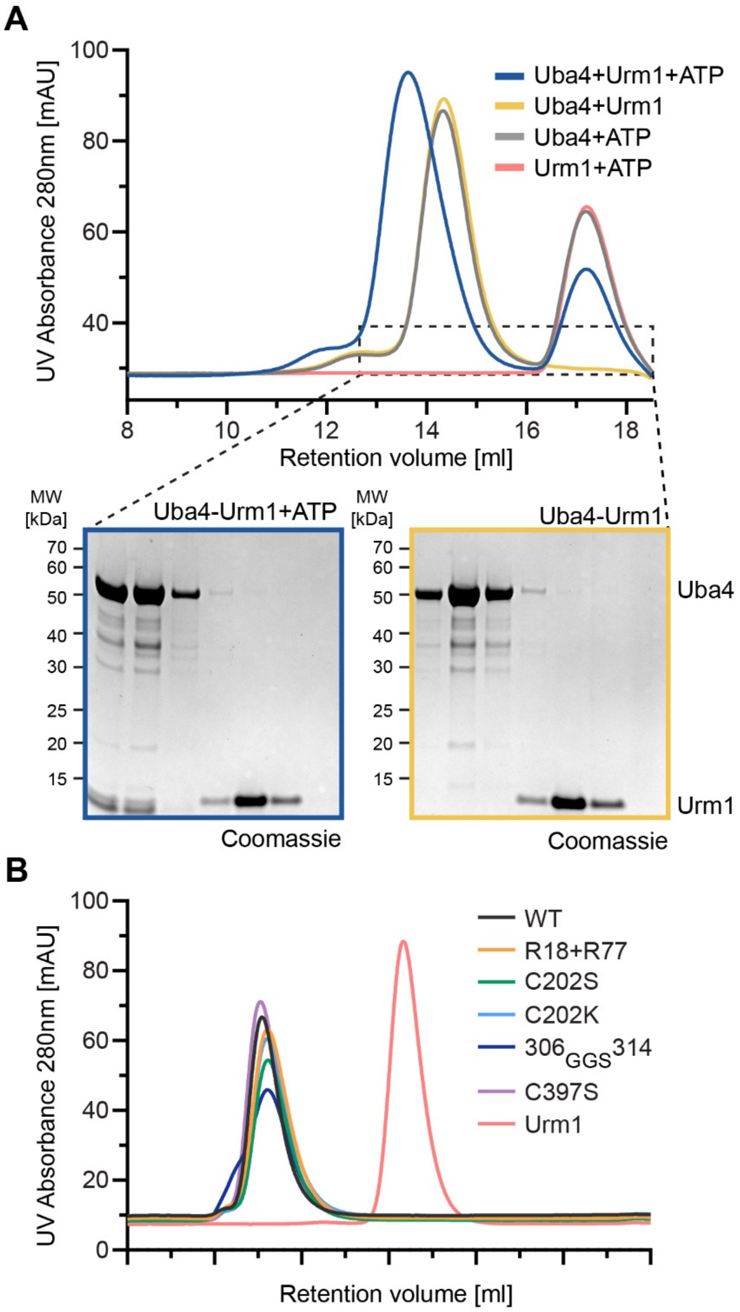

Appendix Figure S4 SEC profiles. (A) Co-migration analyses of *Ct*Uba4 and *Ct*Urm1 using size exlcsuion chromatography. *Ct*Uba4 alone, *Ct*Urm1 alone and *Ct*Uba4 together with *Ct*Urm1 were incubated with ATP and applied on Superdex 200 Increase 10/300 GL column. *Ct*Uba4 together with *Ct*Urm1 without ATP served as control. Fractions containing the complex or the individual proteins were separated on SDS-PAGE. The gel was stained with Coomassie. Complex formation of *Ct*Uba4 and *Ct*Urm1 in the presence of ATP is visible due to the shift of the peak and the appearance of Urm1 in the respective fractions using SDS-PAGE. (B) SEC profiles of purified recombinant proteins, anmely *Ct*Urm1, *Ct*Uba4 WT and indicated mutants. AU, arbitrary units.

Appendix Table S1 Thermostability of CtUba4 proteins. (top) Influence of nucleotides and nucleotide derivatives on the thermostability profile of full-length *Ct*Uba4. Thermostability of selected *Ct*Uba4 mutants. (bottom) Thermostability data (the plotted value corresponds to 50% of the protein being unfolded) for all *Ct*Uba4 mutants in the absence of nucleotides and in the presence of 1 mM AMP or 1 mM ATP, respectively.

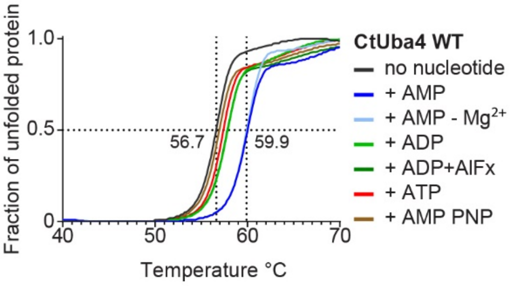

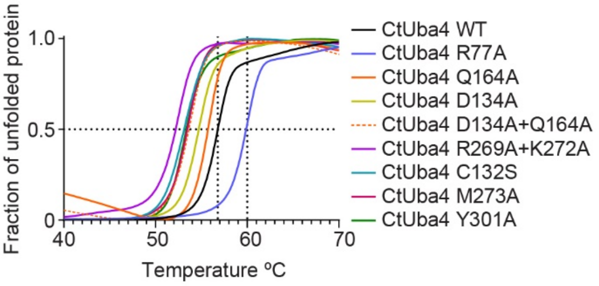

		no nucleotide		AMP			ATP		
ScUba4	CtUba4	mean	SD	mean	SD	Δ	mean	SD	Δ
WT	WT	56.8	0.1	59.9	0.2	3.1	57.4	0.1	0.6
AD	AD	47.1	0.4	53.9	0.3	6.8	49.4	0.5	2.2
$AD_{linker\ 1\text{-}320}$	$AD_{linker\ 1\text{-}315}$	58.1	0.1	61.4	0.2	3.3	58.8	0.1	0.7
$AD_{linker\ 1\text{-}326}$	$AD_{linker\ 1\text{-}321}$	58.2	0.1	61.6	0.1	3.5	59.0	0.1	0.8
C225	C202S	56.9	0.1	60.1	0.2	3.2	57.5	0.1	0.5
C225	C202A	57.4	0.2	60.7	0.2	3.3	57.9	0.1	0.5
C311	C305S	56.4	0.1	59.5	0.2	3.1	56.9	0.1	0.5
C397	C397S	58.8	0.1	61.5	0.2	2.7	59.2	0.1	0.4
C225+C397	C202S+C397S	59.0	0.1	61.6	0.2	2.6	59.5	0.1	0.5
C225+C311	C202S+C305S	56.6	0.1	59.7	0.2	3.1	57.1	0.1	0.5
C225+T193/P214/T215	C202S+K161A/K191A/K192A	57.9	0.1	61.2	0.2	3.3	58.3	0.1	0.3
C311	C305A	56.6	0.1	59.8	0.2	3.2	57.1	0.1	0.5
R49	R18A	56.7	0.1	59.4	0.2	2.7	56.4	0.1	-0.2
R109	R77A	60.0	0.1	60.9	0.2	0.9	59.8	0.1	-0.2
R49+R109	R18A+R77A	59.2	0.1	59.4	0.1	0.2	58.8	0.1	-0.4
D166	D134A	54.7	0.1	59.5	0.2	4.7	55.8	0.2	1.1
Q196	Q164A	55.9	0.2	60.1	0.3	4.2	57.0	0.2	1.1
D176	D144A	57.0	0.2	60.4	0.2	3.4	57.6	0.2	0.6
R275	R269A	54.9	0.1	59.0	0.2	4.0	55.8	0.2	0.9
R275+K278	R269A+K272A	52.6	0.2	57.0	0.2	4.4	53.6	0.2	1.0
Y307	Y301A	53.1	0.1	58.5	0.2	5.4	53.9	0.2	0.8
-	N307A+M308A	57.0	0.2	60.4	0.2	3.4	57.6	0.2	0.5
R398+Y399	R398A+R399A	55.6	0.2	60.1	0.3	4.4	56.9	0.2	1.3
N401+D402	Q401A+D402A	55.4	0.2	60.1	0.3	4.7	56.8	0.2	1.4
D166+Q196	D134A+Q164A	53.9	0.1	59.0	0.2	5.1	55.4	0.2	1.5
-	(306-14) GGS	56.3	0.1	60.2	0.1	3.9	57.3	0.1	1.0
C80	C48S	55.6	0.3	57.4	0.1	1.7	55.5	0.1	-0.1
C164	C132S	53.0	0.2	57.4	0.2	4.3	53.9	0.1	0.8
A178	C146S	55.3	0.2	59.1	0.1	3.7	56.0	0.1	0.7
F212	C189S	56.7	0.1	59.0	0.1	2.3	56.8	0.2	0.1
K278	K272A	54.9	0.1	58.7	0.1	3.8	55.7	0.1	0.8
C225	C202K	57.5	0.1	60.7	0.1	3.3	58.2	0.1	0.7
R171	R139A	55.5	0.1	60.4	0.2	4.8	56.7	0.1	1.2
M279	M273A	53.7	0.2	57.7	0.1	4.1	54.4	0.2	0.7
R171+G192	R139A+Q164A	55.8	0.2	60.8	0.2	5.0	57.4	0.2	1.5
C208+C211	C185S+C188S	56.8	0.2	60.3	0.2	3.5	57.4	0.2	0.6
RHD	RHD	56.4	0.4	56.3	0.3	0.0	55.4	0.3	-0.9
R398+Y399 on RHD	R398A+R399A on RHD	52.6	0.3	52.4	0.4	-0.2	52.1	0.1	-0.6
N401+D402 on RHD	Q401A+D402A on RHD	52.8	0.3	53.0	0.2	0.2	52.2	0.2	-0.6
C397 on RHD	C397S on RHD	63.0	0.3	62.7	0.5	-0.3	62.6	0.3	-0.4

Appendix Table S2 Activity assays of all *Ct*Uba4 mutants *in vitro*. Tabular summary of three assays addressing the ATP hydrolysis by *Ct*Uba4 WT and mutants in the presence of *Ct*Urm1. In the first assay, the amount of released PPi was measured using a fluorometric pyrophosphate assay kit. In the second assay, after PPi hydrolysis to Pi by inorganic phosphatase, Pi was detected using Malachite Green Phosphate Assay kit. In the third assay, the reaction mixture was separated on a SEC column and the area under the peak corresponding to ATP (of UV absorbance at 255 nm) was calculated using the UNICORN 7.0 software. The decrease of the ATP peak area correlates with ATP consumption by Uba4 during Urm1 C-terminus adenylation. Changes in enzymatic activity are indicated as a color gradient from blue to red, normalized to *Ct*Uba4 WT. PPi: pyrophosphate; Pi: inorganic phosphate; SD: standard deviation.

***Sc*Uba4**	***Ct*Uba4**	**PPi [uM]**		**Pi [uM]**		**ATP peak area**
		mean	SD	mean	SD	**(255 nm)**
WT	WT	0.85	0.18	302.4	22.0	24.6
AD	AD	0.58	0.21	193.8	18.2	28.1
AD$_{linker\ 1-320}$	AD$_{linker\ 1-315}$	0.83	0.19	269.2	20.1	nd
AD$_{linker\ 1-326}$	AD$_{linker\ 1-321}$	0.86	0.16	259.3	13.7	nd
AD+RHD	AD+RHD	0.59	0.22	240.7	19.4	30.8
R49	R18A	0.48	0.04	191.9	70.8	27.7
R109	R77A	0.14	0.03	78.0	26.0	32.8
R49+R109	R18A+R77A	0.02	0.18	64.3	37.7	38.1
D166	D134A	0.07	0.27	159.6	60.5	35.7
D166+Q196	D134A+Q164A	-0.04	0.08	72.4	19.0	37.5
C225	C202S	0.83	0.33	253.4	27.8	24.9
C225	C202K	0.77	0.18	179.5	15.0	27.1
C311	C305S	0.89	0.41	398.2	32.8	27.3
C311	C305A	0.93	0.11	294.8	45.6	26.4
C225+C311	C202S+C305S	0.60	0.17	203.6	31.6	22.2
C225+T193/P214/T215	C202S+K161A/K191A/K192A	0.76	0.33	206.6	17.6	nd
C225+C397	C202S+C397S	1.12	0.24	257.0	21.7	25.7
C80	C48S	0.36	0.16	221.6	31.3	33.4
C164	C132S	0.82	0.28	236.0	25.9	25.4
A178	C146S	0.57	0.12	281.7	7.3	26.4
F212	C189S	0.35	0.04	227.4	11.7	29.6
D176	D144A	0.70	0.08	249.2	48.9	24.2
Y307	Y301A	0.98	0.06	265.6	16.2	27.6
R171	R139A	0.75	0.15	178.4	31.0	28.0
R171+Q196	R139A+Q164A	0.33	0.01	101.1	18.8	34.0
Q196	Q164A	0.85	0.13	277.9	16.6	23.5
M279	M273A	0.87	0.25	342.8	21.3	28.2
A313+R314	N307A+M308A	1.33	0.12	402.5	35.0	22.7
	306GGS	0.28	0.10	303.6	50.1	22.3
R275	R269A	0.27	0.26	204.2	16.9	33.7
K278	K272A	0.53	0.09	255.6	33.3	28.7
R275+K278	R269A+K272A	0.28	0.16	202.7	10.4	33.4
C397	C397S	1.59	0.26	407.9	23.3	21.3
R398+Y399	R398A+R399A	1.25	0.11	306.5	52.8	22.1
N401+D402	Q401A+D402A	1.33	0.03	329.1	9.2	21.5
	WT+UrmG111C	-0.29	0.21	20.8	2.0	nd
	no Uba4					**39.0**

Appendix Table S3 Yeast strains used in this study

Strain	Genotype	Background	Source
uba4Δ	*uba4::G418*	S288C	Euroscarf
Uba4_wt	*uba4::3HA-Uba4 HIS*	S288C	this study
Uba4_R49A	*uba4::3HA-Uba4_R49A HIS*	S288C	this study
Uba4_R109A	*uba4::3HA-Uba4_R109A HIS*	S288C	this study
Uba4_R49/109A	*uba4::3HA-Uba4_R49/109A HIS*	S288C	this study
Uba4_D166A	*uba4::3HA-Uba4_D166A HIS*	S288C	this study
Uba4_C208/211A	*uba4::3HA-Uba4_C208/211A HIS*	S288C	this study
Uba4_C225A	*uba4::3HA-Uba4_C225A HIS*	S288C	Leidel et al., 2009
Uba4_C225K	*uba4::3HA-Uba4_C225K HIS*	S288C	this study
Uba4_Y307A	*uba4::3HA-Uba4_Y307A HIS*	S288C	this study
Uba4_C311A	*uba4::3HA-Uba4_C311A HIS*	S288C	this study
Uba4_R275A	*uba4::3HA-Uba4_R275A HIS*	S288C	this study
Uba4_K278A	*uba4::3HA-Uba4_K278A HIS*	S288C	this study
Uba4_M279A	*uba4::3HA-Uba4_M279A HIS*	S288C	this study
Uba4_R275/M279A	*uba4::3HA-Uba4_R275/M279A HIS*	S288C	this study
Uba4_R314A	*uba4::3HA-Uba4_R314A HIS*	S288C	this study
Uba4_C397A	*uba4::3HA-Uba4_C397A HIS*	S288C	Leidel et al., 2009
Uba4_R398/Y399A	*uba4::3HA-Uba4_R398/Y399A HIS*	S288C	this study
Uba4_N401/D402A	*uba4::3HA-Uba4_N401/D402A HIS*	S288C	this study
Uba4_C225/311A	*uba4::3HA-Uba4_C225/311A HIS*	S288C	this study
Uba4_C225/397A	*uba4::3HA-Uba4_C225/397A HIS*	S288C	this study
Uba4_C311/397A	*uba4::3HA-Uba4_C311/397A HIS*	S288C	this study

Appendix Table S4 Results of mass spectrometry analysis of Urm1 conjugation site. Mass spectrometry identification of Urm1-conjugation sites to *Ct*Uba4$_{C202K}$ and CtUba4$_{C202S}$ with peptides containing K-ε-GGH motif of Urm1. Asterisks indicate ambiguous site localisation of K-ε-GGH. #PSMs – number of peptide-to-spectrum matches; m/z – mass to charge ratio of precursor ion.

**Sample**		**Protein**	**Mascot Score**	**Sequence Coverage**	**# Peptides**	**# PSMs**
Uba4C202K_A1		*Ct*Uba4$_{C202K}$	7396	88.06 %	109	361
K-ε-GGH site	Sequence of peptides	Modifications	Mascot Score	# PSMs	Charge	m/z [Da]
K202	KKPPPPSAQTS**k**GEAGIm	K12(GlyGlyHis); M18(Oxidation)	62	4	2	1046.03
	KKPPPPSAQTS**k**GEA	K12(GlyGlyHis)	44	2	3	591.98
	KKPPPPSAQTS**k**GEAGImGPVVGm	K12(GlyGlyHis); M18,M24(Oxidation)	41	4	2	1324.17
	KKPPPPSAQTS**k**GEAGIM	K12(GlyGlyHis)	32	4	3	692.36
	KKPPPPSAQTS**k**GEAGIMGPVVGM	K12(GlyGlyHis)	30	1	2	1308.17
	KKPPPPSAQTS**k**GEAGImGPVVGmm	K12(GlyGlyHis); M18,M24,M25(Oxidation)	20	4	4	699.35
K192	K**k**PPPPSAQTSKGEAGImGPVVGMm	K2(GlyGlyHis); M18,M25(Oxidation)	16	4	3	926.80
**Sample**		**Protein**	**Mascot Score**	**Sequence Coverage**	**# Peptides**	**# PSMs**
Uba4C202K_A2		*Ct*Uba4$_{C202K}$	8344	86.49 %	102	401
K-ε-GGH site	Sequence of peptides	Modifications	Mascot Score	# PSMs	Charge	m/z [Da]
K202	KKPPPPSAQTS**k**GEAGIm	K12(GlyGlyHis); M18(Oxidation)	44	1	3	697.69
	KKPPPPSAQTS**k**GEA	K12(GlyGlyHis)	41	2	3	591.98
	KKPPPPSAQTS**k**GEAGIM	K12(GlyGlyHis)	41	2	3	692.36
	KKPPPPSAQTS**k**GEAGImGPVVGm	K12(GlyGlyHis); M18,M24(Oxidation)	22	1	3	883.11
	KKPPPPSAQTS**k**GEAGImGPVVGM	K12(GlyGlyHis); M18(Oxidation)	17	1	4	658.59
	KKPPPPSAQTS**k**GEAGIMGPVVGMM	K12(GlyGlyHis)	16	1	3	916.13
**Sample**		**Protein**	**Mascot Score**	**Sequence Coverage**	**# Peptides**	**# PSMs**
Uba4C202S_B1		*Ct*Uba4$_{C202S}$	7926	92.34 %	123	356
K-ε-GGH site	Sequence of peptides	Modifications	Mascot Score	# PSMs	Charge	m/z [Da]
K90	**k**VDSLITHLIEINPLPVY	K1(GlyGlyHis)	34	1	3	772.43
K124	DLTPQNAAQII**k**PW	K12(GlyGlyHis)	23	1	3	615.99
K161	VSAASVQ**k**SGQLIVL	K8(GlyGlyHis)	23	1	2	875.99
	VSAASVQ**k**SGQL	K8(GlyGlyHis)	45	2	2	713.38
K191/K192*	**k**KPPPPSAQTSSGEAGIm<br>K**k**PPPPSAQTSSGEAGIm	K1/K2(GlyGlyHis); M18(Oxidation)	41	6	2	1025.50
	**k**KPPPPSAQTSSGEAGImGPVVGMM<br>K**k**PPPPSAQTSSGEAGImGPVVGMM	K1/K2(GlyGlyHis); M18(Oxidation)	30	6	3	907.78
	**k**KPPPPSAQTSSGEAGImGPVVGmm<br>K**k**PPPPSAQTSSGEAGImGPVVGmm	K1/K2(GlyGlyHis); M18,M24,M25(Oxidation)	20	2	3	918.44
	**k**KPPPPSAQTSSGEAGIm<br>K**k**PPPPSAQTSSGEAGIm	K1/K2(GlyGlyHis)	49	4	2	1017.51
	**k**KPPPPSAQTSSGEAGImGPVVGMm<br>K**k**PPPPSAQTSSGEAGImGPVVGMm	K1/K2(GlyGlyHis); M18,M25(Oxidation)	41	8	3	913.11
	**k**KPPPPSAQTSSGEA<br>K**k**PPPPSAQTSSGEA	K1/K2(GlyGlyHis)	29	2	3	578.29
	**k**KPPPPSAQTSSGEAGImGPVVGm<br>K**k**PPPPSAQTSSGEAGImGPVVGm	K1/K2(GlyGlyHis); M18,M24(Oxidation)	28	4	4	652.32
	**k**KPPPPSAQTSSGEAGImGPVVGM<br>K**k**PPPPSAQTSSGEAGImGPVVGM	K1/K2(GlyGlyHis); M18(Oxidation)	26	6	4	648.32
K225	GVAQAGEAI**k**IL	K10(GlyGlyHis)	40	2	2	710.90
	MGVAQAGEAI**k**IL	K11(GlyGlyHis)	26	1	3	517.95
K295	TLDGI**k**SGNPNYVQF	K6(GlyGlyHis)	40	2	2	952.47

**Sample**		**Protein**	**Mascot Score**	**Sequence Coverage**	**# Peptides**	**# PSMs**
Uba4C202S_B2		*Ct*Uba4$_{\text{C202S}}$	7236	91.67%	117	330
K-ε-GGH site	Sequence of peptides	Modifications	Mascot Score	# PSMs	Charge	m/z [Da]
K10	S**k**EEIERY	K2(GlyGlyHis)	20	1	3	435.55
K90	**k**VDSLITHLIEINPLPVY	K1(GlyGlyHis)	21	1	3	772.43
K124	DLTPQNAAQII**k**PW	K12(GlyGlyHis)	34	1	3	615.99
K161	VSAASVQ**k**SGQL	K8(GlyGlyHis)	37	1	2	713.38
K191/K192*	**k**KPPPPSAQTSSGEAGImGPVVGm<br>K**k**PPPPSAQTSSGEAGImGPVVGm	K1/K2(GlyGlyHis);<br>M18,M24(Oxidation)	34	2	3	869.42
	**k**KPPPPSAQTSSGEAGIMGPVVGM<br>K**k**PPPPSAQTSSGEAGIMGPVVGM	K1/K2(GlyGlyHis)	18	2	4	644.32
	**k**KPPPPSAQTSSGEAGIm<br>K**k**PPPPSAQTSSGEAGIm	K1/K2(GlyGlyHis); M18(Oxidation)	44	4	2	1025.51
	**k**KPPPPSAQTSSGEAGImGPVVGMM<br>K**k**PPPPSAQTSSGEAGImGPVVGMM	K1/K2(GlyGlyHis); M18(Oxidation)	40	2	3	907.78
	**k**KPPPPSAQTSSGEA<br>K**k**PPPPSAQTSSGEA	K1/K2(GlyGlyHis)	27	4	3	578.30
	**k**KPPPPSAQTSSGEAGIM<br>K**k**PPPPSAQTSSGEAGIM	K1/K2(GlyGlyHis)	27	2	3	678.68
	**k**KPPPPSAQTSSGEAGImGPVVGM<br>K**k**PPPPSAQTSSGEAGImGPVVGm	K1(GlyGlyHis); M18(Oxidation)	20	6	3	864.10
K225	GVAQAGEAI**k**IL	K10(GlyGlyHis)	43	2	2	710.90
	MGVAQAGEAI**k**IL	K11(GlyGlyHis)	32	1	3	517.95
	GVAQAGEAI**k**ILVSQL	K10(GlyGlyHis)	25	1	3	616.68
K295	TLDGI**k**SGNPNYVQF	K6(GlyGlyHis)	42	2	2	952.47
**Sample**		**Protein**	**Mascot Score**	**Sequence Coverage**	**# Peptides**	**# PSMs**
Uba4C202S_C1		*Ct*Uba4$_{\text{C202S}}$	5800	93.24%	138	260
K-ε-GGH site	Sequence of peptides	Modifications	Mascot Score	# PSMs	Charge	m/z [Da]
K161	VSAASVQ**k**SGQL	K8(GlyGlyHis)	37	1	2	713.376
	VSAASVQ**k**SGQLIVL	K8(GlyGlyHis)	27	2	2	875.994
K191/K192*	**k**KPPPPSAQTSSGEA<br>K**k**PPPPSAQTSSGEA	K1/K2(GlyGlyHis)	30	2	3	578.292
	**k**KPPPPSAQTSSGEAGImGPVVGm<br>K**k**PPPPSAQTSSGEAGImGPVVGm	K1/K2(GlyGlyHis);<br>M18,M24(Oxidation)	22	4	3	869.427
	**k**KPPPPSAQTSSGEAGImGPVVGmm<br>K**k**PPPPSAQTSSGEAGImGPVVGmm	K1/K2(GlyGlyHis);<br>M18,M24,M25(Oxidation)	46	4	3	918.440
	**k**KPPPPSAQTSSGEAGIm<br>K**k**PPPPSAQTSSGEAGIm	K1/K2(GlyGlyHis); M18(Oxidation)	42	2	2	1025.505
	**k**KPPPPSAQTSSGE<br>K**k**PPPPSAQTSSGE	K1/K2(GlyGlyHis)	18	1	3	554.613
K295	TLDGI**k**SGNPNYVQF	K6(GlyGlyHis)	27	1	3	635.315
**Sample**		**Protein**	**Mascot Score**	**Sequence Coverage**	**# Peptides**	**# PSMs**
Uba4C202S_C2		*Ct*Uba4$_{\text{C202S}}$	6064	96.17%	147	262
K-ε-GGH site	Sequence of peptides	Modifications	Mascot Score	# PSMs	Charge	m/z
K161	VSAASVQ**k**SGQL	K8(GlyGlyHis)	36	1	2	713.37
K191/K192*	**k**KPPPPSAQTSSGEAGIm<br>K**k**PPPPSAQTSSGEAGIm	K1/K2(GlyGlyHis); M18(Oxidation)	56	2	2	1025.50
	**k**KPPPPSAQTSSGE<br>K**k**PPPPSAQTSSGE	K1/K2(GlyGlyHis)	38	4	2	831.41
	**k**KPPPPSAQTSSGEAGImGPVVGmm<br>K**k**PPPPSAQTSSGEAGImGPVVGmm	K1/K2(GlyGlyHis);<br>M18,M24,M25(Oxidation)	35	4	3	918.44
	**k**KPPPPSAQTSSGEAGImGPVVGm<br>K**k**PPPPSAQTSSGEAGImGPVVGm	K1/K2(GlyGlyHis);<br>M18,M24(Oxidation)	30	4	4	652.32
K295	TLDGI**k**SGNPNYVQF	K6(GlyGlyHis)	25	1	3	635.31
