# Supplementary material for: Molecular basis for the bifunctional Uba4–Urm1 sulfur‐relay system in tRNA thiolation and ubiquitin‐like conjugation
Source: EMBO J. 2020 Sep 9;39(19):e105087. doi: 10.15252/embj.2020105087 (PMC7527816; doi:10.15252/embj.2020105087)
Supplement: Supplementary file 2 — Expanded View Figures PDF [file EMBJ-39-e105087-s002.pdf]

Expanded View Figures

A

Ct Proteins	Calculated MW of a monomer [kDa]	MW determined by RALS/LALS [kDa]
Uba4 FL WT	47.95	96.66
Uba4 AD WT	30.95	65.64
Uba4 RHD WT	14.22	28.79
Uba4 RHD R398A+R399A	14.05	14.64 / 12.42
Urm1	12.48	13.30
Uba4 FL WT+Urm1	60.43 (1:1)*	116.50
Uba4 FL C202S+C397S	47.92	97.84
Uba4 FL C202S+C397S +Urm1	60.40 (1:1)*	123.60

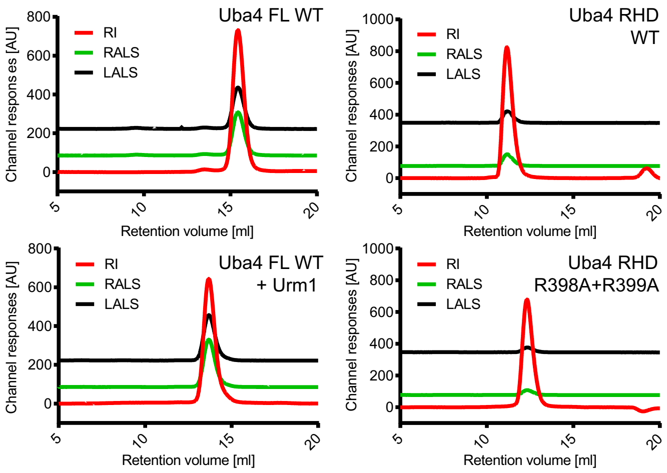

B

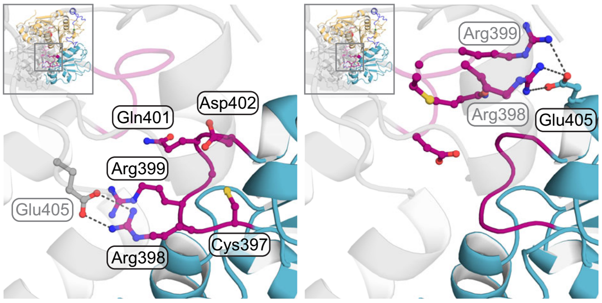

C

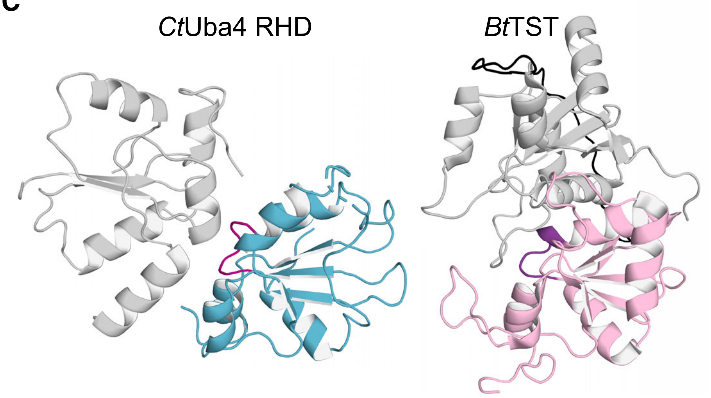

Figure EV1. *Ct*Uba4 FL, AD, and RHD are dimers in solutions.

A Tabular summary of static light-scattering (SLS) experiments. Calculated molecular weight (MW) of the indicated proteins or protein complexes is compared to experimentally determined MWs. *For the Uba4–Urm1 complex, the MW is calculated for a 1:1 protein ratio. Selected graphs from SLS measurements are presented (right). Profiles of refractive index (RI, red), right-angle light scattering (RALS, green), and low-angle light scattering (LALS, black) are shown. MW: molecular weight; FL: full length; WT: wild type, AU: arbitrary units.

B Close-up view of the *Ct*Uba4 RHD active loop (purple) with ball-and-stick representation of amino acids. The second RHD molecule (grey, transparent) is shown to visualize the dimer interface. Hydrogen bonds are indicated as dashed lines.

C Structural comparison of the RHD active loop localization in relation to the dimeric interface of the domain in *Ct*Uba4 (cyan) and *Bos taurus* thiosulfate sulfurtransferase (*Bt*TST; pink; PDB ID 1RHD).

Figure EV2. Structural details of apo Uba4.

A Close-up view of the *Ct*Uba4 P-loop-like region and the nucleotide-binding site. Amino acids required for ATP binding are shown as ball-and-stick model colored according to atom type. The ATP molecule (green) was modeled based on the structural similarity to the ATP-bound *Ec*MoeB-MoaD (PDB ID: 1JWA).

B Close-up of the *Ct*Uba4 linker (aa 286–321) showing the refined $2F_o$-F_c electron-density map contoured at 1.0σ. Coordination of the zinc site is highlighted.

C Cartoon representation of *Ct*Uba4 and the respective structural model of *Sc*Uba4 highlighting selected mutated amino acid residues shown as black ball-and-stick model.

D Interaction analysis of WT and mutated *Ct*Uba4 with GST-*Ct*Urm1 by GST pull-down in the absence and presence of 1 mM ATP. Input samples (left) and samples after GST pull-down (right) are resolved by SDS–PAGE and visualized with Coomassie stain.

▶

© 2020 The Authors

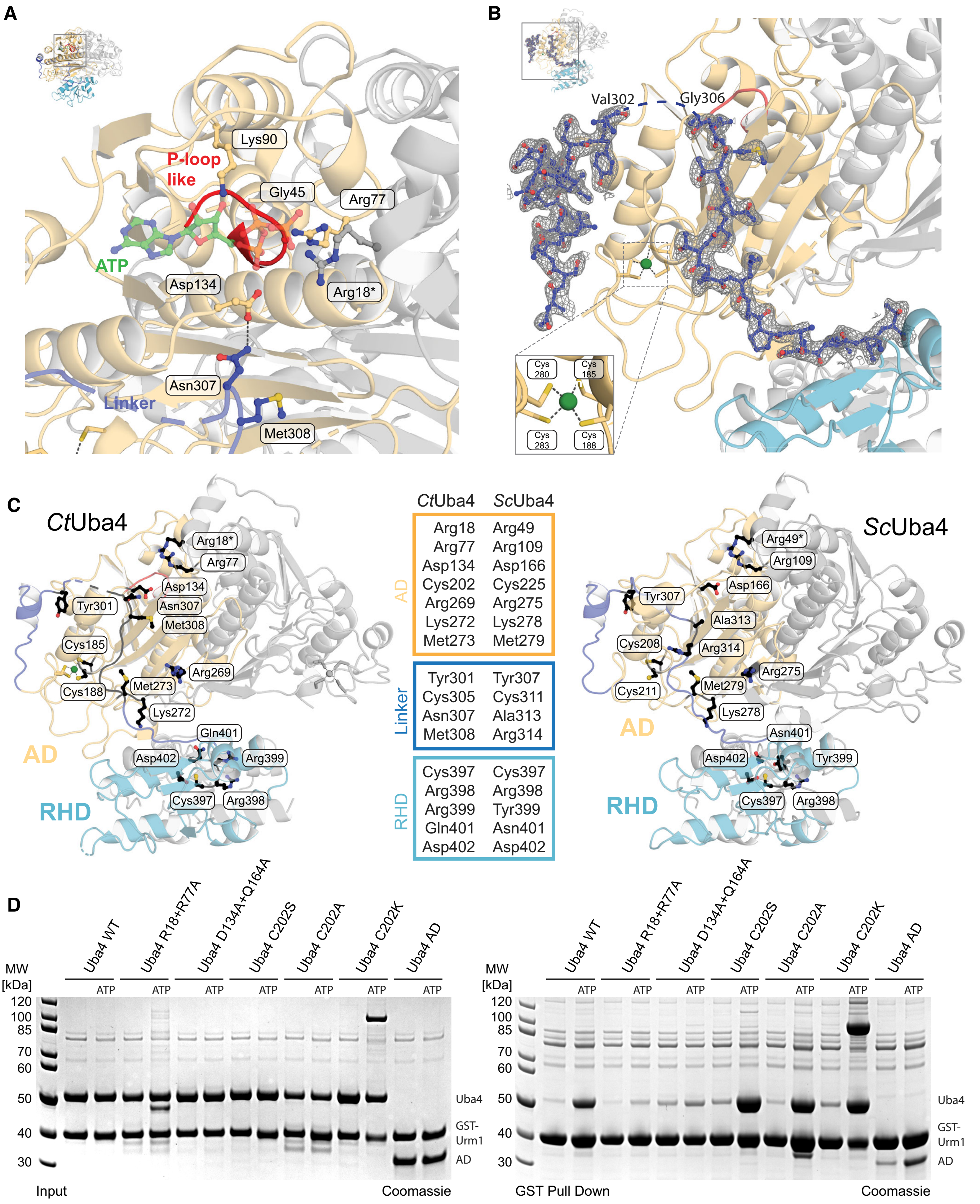

Figure EV2.

 © 2020 The Authors

Figure EV3. Structural analyses of the Uba4–Urm1 complex.

A Refined $2F_o$-F_c electron-density maps contoured at 1.0σ for apo *Ct*Uba4 (left) and *Ct*Urm1-bound *Ct*Uba4 (middle). Densities of ADs (grey), RHD (cyan), and Urm1 (salmon) are labeled. Cartoon model with transparent surface representation of the $Uba4_{C202K}$-Urm1 complex structure highlighting the detailed B-factor distribution (right).

B Close-up of the active site of Uba4 linked to the C-terminus of Urm1 (bottom left) showing refined densities of the Urm1 C-terminus (red), the crossover loop of Uba4 (brown), and Lys202 (green) at 1σ (top left). Comparison between refined $2F_o$-F_c maps of individual regions with F_o-F_c omit maps after omitting the respective parts of the model.

© 2020 The Authors

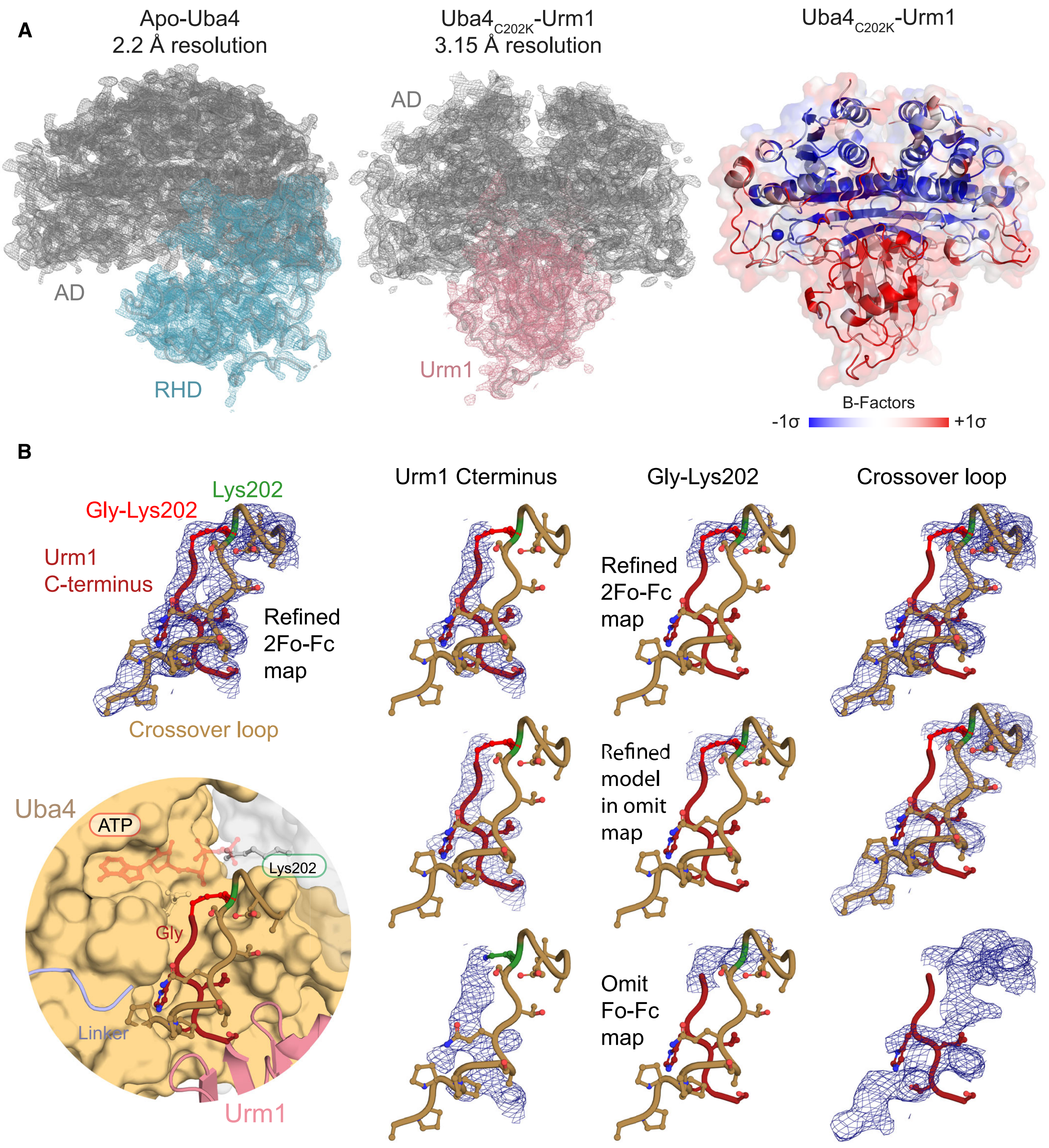

Figure EV3.

 © 2020 The Authors

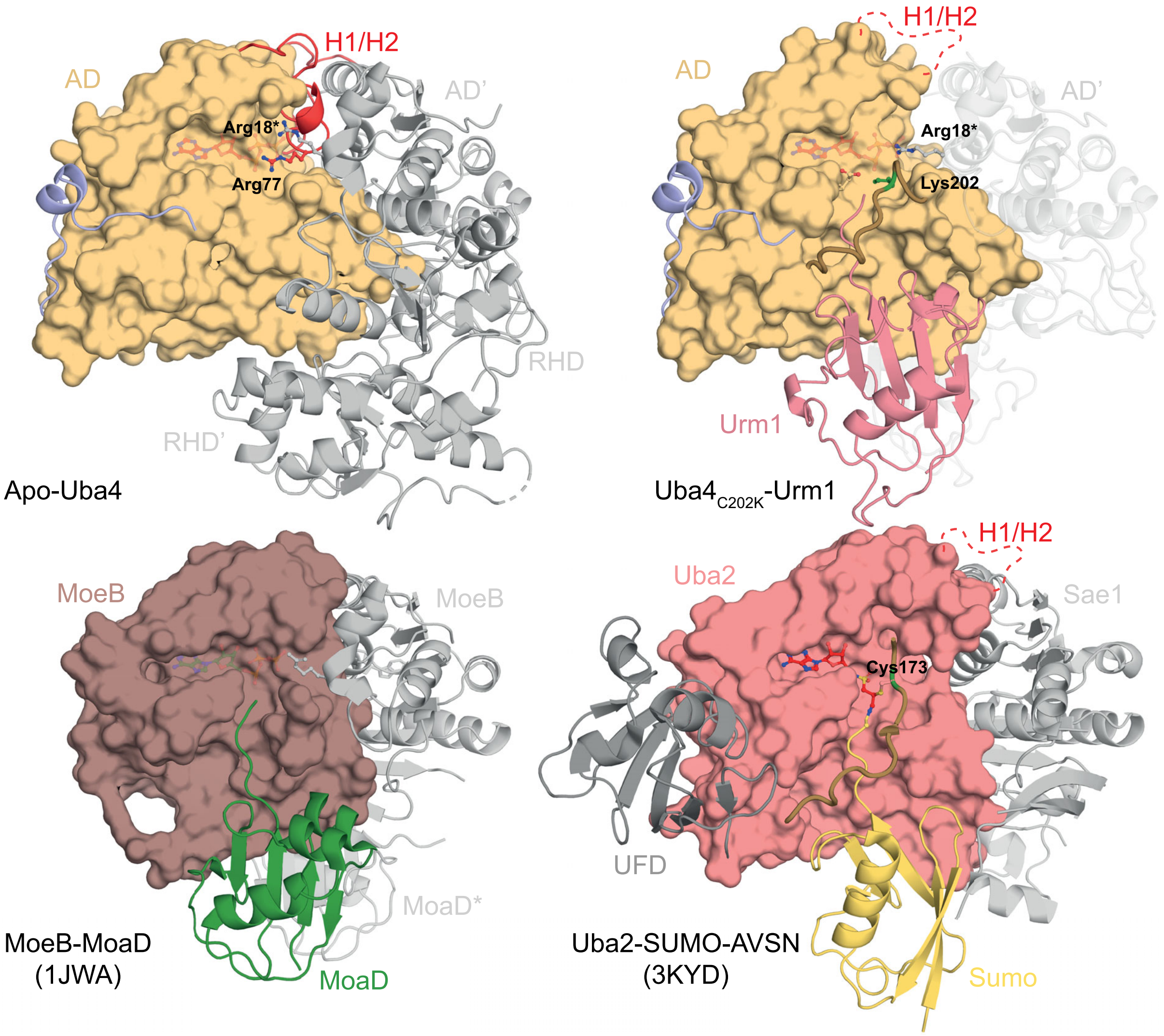

Figure EV4. Structural comparison of the Uba4–Urm1 complex.

Comparison of the crystal structures of apo and Urm1-bound *Ct*Uba4 (top) with the prokaryotic molybdenum-cofactor-biosynthesis protein MoeB bound to its substrate MoaD (PDB ID: 1JWA) and the eukaryotic SUMO E1 enzyme bound to its chemically modified substrate SUMO-AVSN (PDB ID: 3KYD). E1-like adenylation domains are shown as surface. The remaining domains or interacting proteins and bound UBL or SCP are shown as cartoon representation. The crossover loop containing the active cysteine (green) is highlighted. The H1/H2 helix that undergoes remodeling upon Urm1 modification is shown in red. The ATP molecule (red) is shown as ball-and-stick model.

Figure EV5. Functional analysis of the Uba4 active-site cysteine.

- A Analysis of covalent adduct formation between *Ct*Uba4 WT or C202K and carboxylated (–OH) or thiocarboxylated (–SH) *Ct*Urm1 in the presence or absence of *tert*-Butyl hydroperoxide (TBH). HA: Hydroxylamine; DTT: 1,4-Dithiothreitol; TCEP: Tris(2-carboxyethyl) phosphine; ATP: Adenosine triphosphate.
- B tRNA thiolation status of different *Sc*Uba4 mutant yeast strains analyzed by APM-gel-retardation assay and Northern blot. APM: ([*N*-Acryloyl-amino]phenyl)mercuric chloride. All residue numbering follows the *Ct*Uba4 sequence, but the respective *Sc*Uba4 numbering is added in subscript.
- C Analysis of covalent adduct formation between WT or mutated *Ct*Uba4 and carboxylated (–OH) or thiocarboxylated (–SH) *Ct*Urm1 in the presence of ATP and TBH, respectively.
- D Analysis of covalent adduct formation between WT or mutated *Ct*Uba4 and *Ct*Urm1-COSH in the presence of TBH.
- E Left: Viability analysis of *ScUba4* mutant yeast in response to rapamycin. Right: Analysis of tRNA thiolation status in *ScUba4* mutant yeast strains by APM-gel-retardation assay and Northern blotting. Addition of APM to the gel retards the migration of thiolated tRNAs and allows their visualization. APM: ([*N*-Acryloyl-amino]phenyl)mercuric chloride. All residue numbering follows the *Ct*Uba4 sequence, but the respective *Sc*Uba4 numbering is added in subscript.
- F Schematic overview of various possible conjugation reaction routes of active-site substitutions upon Urm1-COSH oxidation. Main reaction path is indicated by a solid line and side reaction by a dashed line, respectively.

▶

© 2020 The Authors

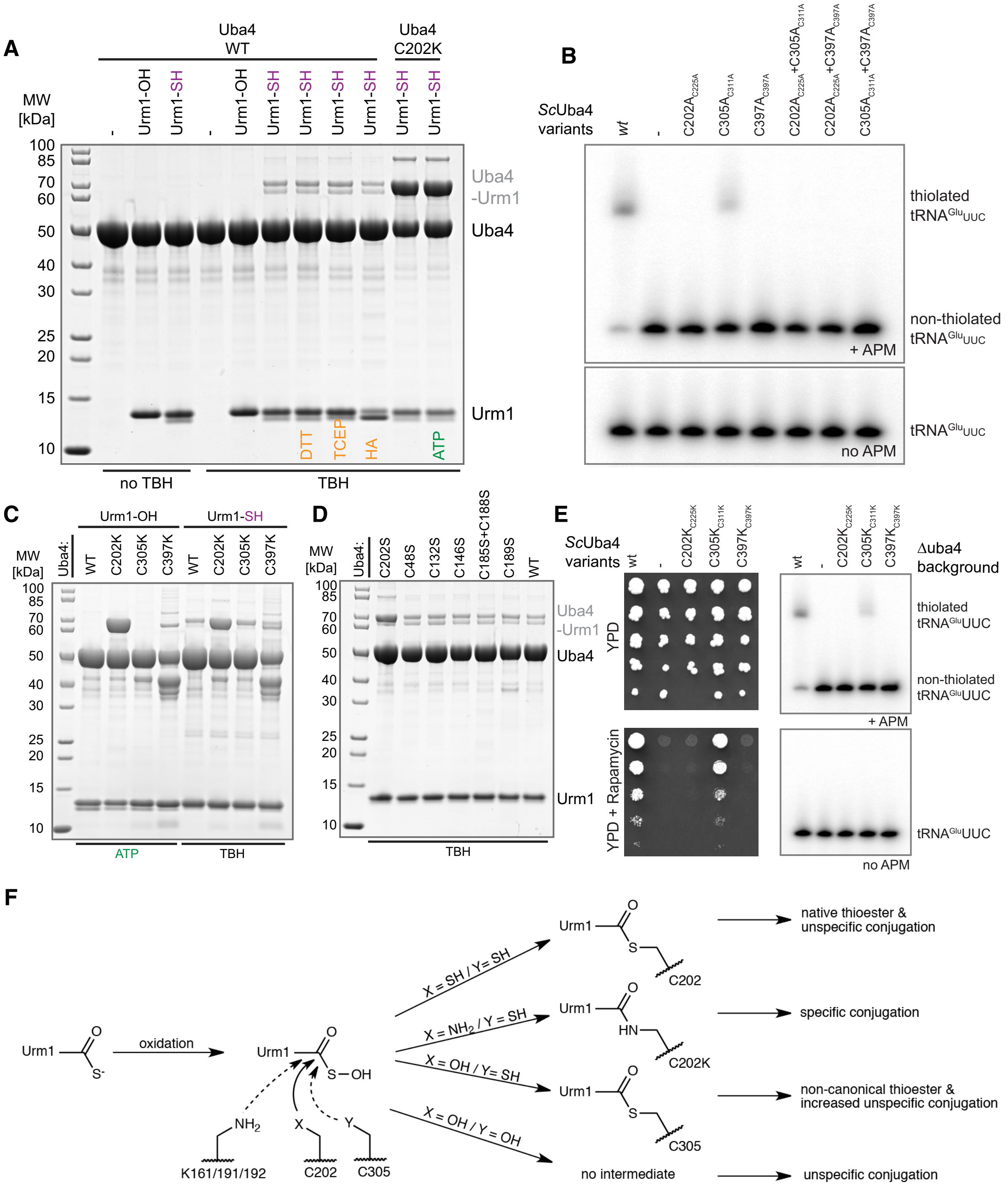

Figure EV5.

 © 2020 The Authors
